# Removal of Trithiocarbonyl End Group of RAFT-Polymerized Poly(stearyl acrylate) and Effect of the End Group on Thermal and Structural Properties

**DOI:** 10.3390/polym13234169

**Published:** 2021-11-28

**Authors:** Eri Oishi, Masumi Takamura, Tatsuhiro Takahashi

**Affiliations:** 1 Department of Organic Materials Science, Graduated School of Organic Materials Science, Yamagata University, 4-3-16 Jonan, Yonezawa 992-8510, Yamagata, Japan; tky45776@st.yamagata-u.ac.jp; 2 Open Innovation Platform, Yamagata University, 4-3-16 Jonan, Yonezawa 992-8510, Yamagata, Japan; masumi_takamura@yz.yamagata-u.ac.jp

**Keywords:** RAFT polymerization, poly(stearyl acrylate), poly(octadecyl acrylate), semi-crystalline polymer, side chain crystalline polymer, end group effect

## Abstract

The effect of a long alkyl end group on the thermal and structural properties of RAFT (reversible addition-fragmentation chain transfer)-polymerized poly(stearyl acrylate) (PSA) was investigated. RAFT-polymerized PSA was prepared using 2-cyano-2-[(dodecylsulfanylthiocarbonyl) sulfanyl] propane (CDTP) with long alkyl group as a chain transfer agent and azobisisobutyronitrile (AIBN) as an initiator. The RAFT polymerization resulted in the polymerized structure having trithiocarbonyl (TTC) at one end and isobutyronitrile at the other end. RAFT-polymerized PSA was prepared with two different molecular weights. The TTC end group was replaced by isobutyronitrile using radical reaction with AIBN through optimization of the conditions, which resulted in isobutyronitrile at both ends. The effect of the end group on the thermal and structural properties was investigated using differential scanning calorimetry and X-ray diffraction, and the results indicated that the long alkyl group from TTC lowers the melting point and semi-crystalline structure in the case of low molecular weight PSA.

## 1. Introduction

End group modification of polymers has received considerable attention, especially for polymers with specific functional groups as side chains, such as polyacrylate and polyacrylamide, with an aim to further improve functionalization toward various applications [1,2,3,4]. There are two end groups for linear polymers without branching structures, and the influence of the end groups is significant when the molecular weight of the polymer is lower. Reports related to polymers with narrow molecular weight distribution produced by living radical polymerization has recently increased in a drive to enhance the effect of end groups with reproducibility and uniformity.

Although there are various methods for living radical polymerization, reversible addition-fragmentation chain transfer (RAFT) polymerization has been frequently utilized for various monomers, such as conjugated vinyl monomers (i.e., acrylate) and non-conjugated monomers (i.e., vinyl acetate) by optimization of the chain transfer agents (CTAs) [5,6,7,8,9]. There have been many previous reports on RAFT-polymerized polymers with trithiocarbonyl (TTC) groups as the one end group, which was modified in various ways, including by using nucleophile agents [10,11,12,13], oxidation agents [14,15], and protonation [10,13,16].

Among the various end modifications, the most frequently utilized method is the use of radical initiators [13,17,18,19,20]. The chemical reaction proceeds with the dozens of times the initiator (based on moles) for polymerization is added and reacted, which results in cleavage of the C-S bond between the polymer end and the TTC group, and the further addition reaction of the initiator radical. The end group, after the modification with this method, is determined by the chemical structure of the applied radical initiators. Therefore, various radical initiators have been utilized for the modification of end groups produced by RAFT polymerization, such as azo radical initiators [17,18,19,20] and peroxide radical initiators [18,20].

The polymer crystallinity of acrylate polymers, either amorphous or semi-crystalline, is determined by the number of alkyl groups in the side chain. An acrylate polymer with longer alkyl groups of more than 6 carbons as side chains is semi-crystalline and has strong hydrophobic properties and a low glass transition temperature. Among these polymers, poly(stearyl acrylate) (PSA) has been utilized for applications such as the surface modification of polyethylene [21], control of polymer deterioration [22], and as a positive temperature coefficient (PCT) thermistor [23]. The crystalline region forms as the side chains line up in parallel for a semi-crystalline polymer with a side chain, which is different from that of a semi-crystalline main chain polymer, such as polyethylene. The melting point of the semi-crystalline polymer increases with the molecular weight; however, there have been no reports on the effect of end groups on the thermal properties and crystalline structure of such polymers.

In this paper, PSA, a semi-crystalline polymer with side chains, was synthesized through RAFT polymerization. The complete removal of the one end group of CTA was examined under various conditions for optimization. The effect of the end group on the crystalline properties was investigated. 2-Cyano-2-[(dodecylsulfanylthiocarbonyl) sulfanyl] propane (CDTP), a trithiocarbonyl (TTC) type CTA with a long alkyl chain of 12 carbons, was used as the CTA for the RAFT polymerization. Azobisisobutyronitrile (AIBN) was used as a radical initiator to remove the TTC end groups. The exact amount of TTC removed was carefully analyzed using nuclear magnetic resonance spectroscopy (NMR), elemental analysis, and matrix assisted laser deposition/ionization time of flight mass spectrometry (MALDI-TOF-MS). The effect of TTC removal on the crystallinity was examined using differential scanning calorimetry (DSC) together with analysis of the degree of crystallinity from X-ray diffraction measurements. PSAs with two different molecular weights were synthesized and the influence of the end group on the molecular weight was also examined.

## 2. Materials and Methods

### 2.1. Materials

Stearyl acrylate (SA) was obtained from Tokyo Chemical Industry (Tokyo, Japan), and the inhibitor was removed by recrystallization in methanol. CDTP was obtained from Fujifilm Wako Pure Chemical Co. (Osaka, Japan). AIBN and dehydrated toluene were obtained from Kanto Chemical Co., Inc. (Tokyo, Japan). AIBN was purified by recrystallization in ethanol.

### 2.2. Synthesis of PSA by RAFT Polymerization

**Scheme 1:**
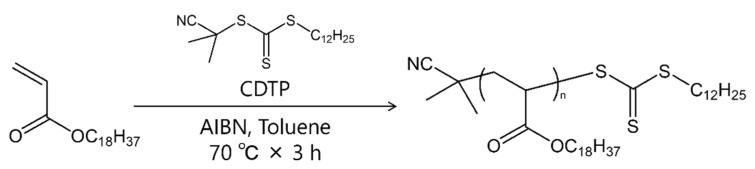
Synthesis of PSA by RAFT polymerization using CDTP as chain transfer agent.

Scheme 1 shows the synthesis route for RAFT-polymerized PSA with CDTP as the CTA, and Table 1 shows the properties of the polymerized PSA. The details of synthesis are as follows.

PSA4.9k: SA (2.94 g, 9.07 mmol), CDTP (210.4 mg, 0.61 mmol), and recrystallized AIBN (34.6 mg, 0.21 mmol) were dissolved in 4.5 mL of anhydrous toluene in a 30 mL Schlenk flask. Three freeze-pump-thaw cycles were performed, after which the flask was filled with nitrogen and removed from the vacuum line. The solution was stirred at 70 °C for 3 h and the reaction was then quenched. The reacted solution was reprecipitated in methanol, followed by filtration. The polymer was dried under vacuum after three precipitation/filtration cycles to obtain the product as a yellow powder.

PSA17k: SA (0.98 g, 3.01 mmol), CDTP (10.8 mg, 0.03 mmol), and recrystallized AIBN (1.6 mg, 0.01 mmol) were dissolved in 1.5 mL of anhydrous toluene in a 30 mL Schlenk flask. Three freeze-pump-thaw cycles were performed, after which the flask was filled with nitrogen gas and removed from the vacuum line. The solution was stirred at 70 °C for 3 h, and the reaction was then quenched. Purification of the polymer was the same as that for PSA4.9k, to obtain the product as a pale-yellow powder.

### 2.3. Removal of TTC End Group from RAFT Polymerized PSA and Modifying Isobuthironitrile End Group

**Scheme 2:**
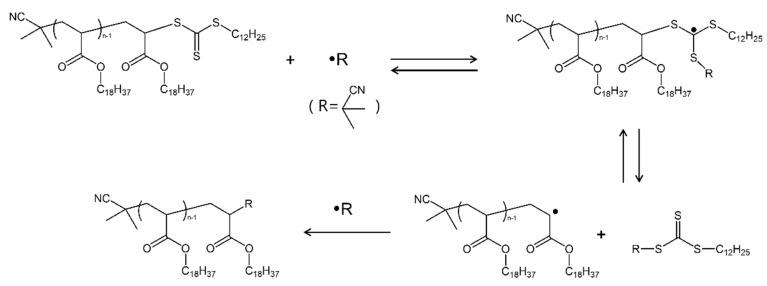
Removal of TTC end group and modifying isobutyronitrile end group by conventional radical reaction with AIBN as radical resource.

Scheme 2 shows removal of TTC and modifying isobuthironitrile end group reactions. The synthesized PSA was dissolved in toluene (100 mg/mL), and AIBN was added to the solution in a PSA:AIBN ratio of 1:30. Three freeze-pump-thaw cycles were performed, after which the flask was filled with nitrogen gas. The reaction of TTC end group removal from PSA4.9k was conducted with various temperatures and reaction times: 90 °C for 2 h, 80 °C for 2.5 h (same condition as [17]), 70 °C for 16 h, and 65 °C for 24 h. The reaction of TTC removal from PSA17k was performed at 70 °C for 16 h. The reacted solution was precipitated in acetone, followed by filtration, and drying under vacuum, to obtain each product after the reactions.

### 2.4. Polymer Characterization

^1^H-NMR (JNM-EC500, JEOL, Tokyo, Japan; 500 MHz) was used to determine monomer conversion, the number average of molecular weight, and the TTC removal rate. CDCl_3_ was used as the solvent for NMR analysis, and the chemical shift was calibrated using residual CHCl_3_ (at 7.26 ppm) as the internal standard. Size exclusion chromatography (SEC; GULLIVER 1500, JASCO, Tokyo, Japan) analysis was performed using a chromatograph equipped with a pump, an absorbance detector (UV, λ = 254 nm), and three polystyrene gel columns, based on a conventional calibration curve using polystyrene standards. The eluent was tetrahydrofuran (THF) at a flow rate of 1.0 mL/min at 40 °C. Elemental analysis (EA; CHN and S) was performed with a CHNS/O Elemental Analyzer 2400 II (Perkin-Elmer, Waltham, MA, USA) calibrated using stearic acid, sulfanilamide, sulfathiazole, 1,3-diphenylthiourea (Kishida Chemical Co., Ltd., Elemental analysis standard, Japan) and cystine (Perkin-Elmer, Waltham, MA, USA). Matrix-assisted laser desorption ionization time-of flight mass spectrometry (MALDI-TOF MS) analysis was performed on a JMS-3000 Linear TOF spectrometer (JEOL Ltd., Japan) at an acceleration voltage of 20 kV in the positive linear mode. External mass calibration was performed using a PSt standard (Mn = 5000). Trans-2-[3-(4-tert-buthylphenyl)-2-methyl-2-propanylidene]malononitrile (DCTB) was used as the matrix and sodium trifluoroacetate as the cationization agent. The polymer (10 mg/mL), matrix (20 mg/mL), and cationization agent (1 mg/mL) were dissolved in THF, and these solutions were mixed at a volume ratio of 1:5:1. 1 µL of the mixed solution was deposited on a MALDI sample plate, and the spots were dried in air at room temperature. Thermogravimetric analysis (TGA) was conducted using a TG-DTA8122 with Smart loader analyzer (Rigaku, Japan) in a nitrogen atmosphere, with calibration using indium. All samples were weighed (8–9 mg) and then heated to 500 °C at 5 °C/min. Differential scanning calorimetry (DSC) analysis was conducted using a calorimeter (DSC Q200, TA Instruments, Tokyo, Japan) with a refrigerated cooling system (RCS, TA Instruments Japan) that was calibrated with sapphire for calibration of the cell resistance and thermal capacity, and with indium for the cell constant and temperature calibration. All samples were weighed (2–3 mg), heated to 80 °C at 5 °C/min followed by holding at 80 °C for 10 min, and then cooled to −30 °C at 5 °C/min followed by holding at −30 °C for 10 min. The heating and cooling cycle was conducted 2 times. All values reported in this work were taken from the first cooling and second heating cycles. X-ray diffraction (XRD) measurements were performed at the BL03XU beamline, Spring-8, Japan.

## 3. Results and Discussion

### 3.1. Optimization for TTC End Group Removal and Modification with the Isobutyronitrile End Group by Radical Reaction with AIBN

**Figure 1:**
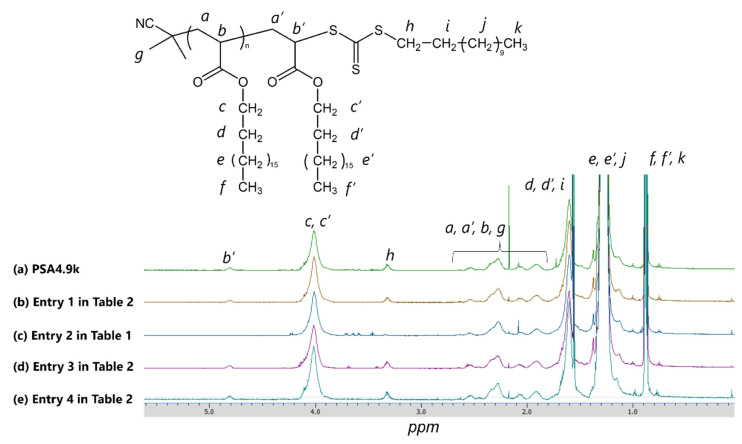
^1^H-NMR spectra for (**a**) RAFT-polymerized PSA 4.9k in Table 1; (**b**–**e**) TTC removal from PSA 4.9k conducted under various conditions.

Table 2 shows the various conditions for removal of the TTC end group from RAFT-polymerized PSA and then modification with the isobutyronitrile end group. The TTC removal rate was calculated from ^1^H-NMR measurements. Figure 1 shows ^1^H-NMR spectra for the polymers reacted under various conditions (Entries 1–4) and the PSA4.9k precursor. The peaks of focus are *b*’ and *h*, where peak *h* is S-CH_2_- in TTC, peak *b*’ is S-CH- the SA unit next to TTC, and the integral ratio of *h*:*b*’ = 2:1 for the PSA 4.9k precursor. Therefore, if all TTC was removed, then the peaks of *b*’ and *h* should disappear. Perrier et al. [17] reported the successful removal of the TTC end group from RAFT-polymerized poly(methyl methacrylate) (PMMA) and modification with various end groups by radical reaction with azo-type initiators in a initiator:PMMA ratio of 20:1 at 80 °C for 2.5 h. Therefore, 30 eq. AIBN was taken in the present experiment under the condition of 80 °C for 2.5 h (Table 2 and Figure 1, Entry 3) to obtain PSA without TTC. Although the AIBN decomposition rate at 80 °C for 2.5 h was 72.2%, the TTC end groups were not removed. We then attempted the procedure using a higher temperature of 90 °C for 3 h (Table 3 and Figure 1, Entry 4), by which the AIBN decomposition rate was 99.5%; therefore, more AIBN was decomposed under this condition. However, the TTC removal reaction did not occur.

**Figure 2:**
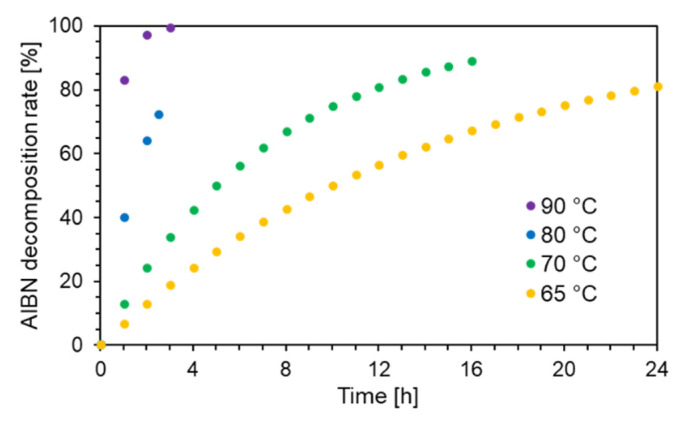
Calculated time-decomposition rate for AIBN.

The time-decomposition rate for AIBN at 65–90 °C was calculated and plotted (Figure 2). At 90 °C, the 1 h decomposition rate was over 80%, which indicates that radicals are immediately generated, although radical deactivation is also very fast. At 80 °C, the 1 h later decomposition rate was 40%; the decomposition rate was less than that at 90 °C. Although it should be very fast, there was concern that radical deactivation would occur before the radicals reacted with TTC. In addition, the radical concentration was very high initially, but rapidly decreased with time.

Entries 1 and 2 in Table 2 show the TTC removal reaction conducted at 65 °C for 24 h and 70 °C for 16 h, respectively. These AIBN decomposition rates per unit time were lower than those at 90 °C and 80 °C; however, radical generation was sustainable for a long time. Entry 2 in Figure 1 shows that the *b*’ peak almost disappeared and the h peak was decreased. The reason why only the h peak remained was that the removed TTC (the structure is the same as CDTP) would be included in purified PSA after the TTC removal reaction (Entry 2). The TTC removal rate was calculated from the ratio of (c+c’) and *b*’ integrals from ^1^H-NMR measurements. The TTC removal rate in Entry 2 was 88%, which demonstrates that the condition of sustainable radical generation for a long time is necessary for the TTC removal reaction by AIBN with the RAFT-polymerized PSA. The TTC removal reaction was then conducted at 65 °C for 24 h (Entry 1); this condition continued to generate radicals for a longer time than Entry 2. However, Entry 1 in Figure 1 shows TTC remained (i.e., peak *b*’ and *h*) more so than Entry 2, and the TTC removal rate was only 32%. Radicals continued to be gradually generated at 65 °C for 24 h; however, the concentration of radicals per unit time was not sufficient to remove the TTC end group from RAFT-polymerized PSA. The removal of TTC from PSA17k was performed at 70 °C for 16 h only (Entry 5). PSA17k has higher molecular weight, so that the difference before and after reaction could not be determined because the proton peaks of *b*’ and *h* were very weak. Therefore, TTC removal from PSA17k was conducted using the most suitable condition for TTC removal from PSA4.9k.

### 3.2. Polymer Characterization

#### 3.2.1. Size Exclusion Chromatography (SEC)

**Figure 3:**
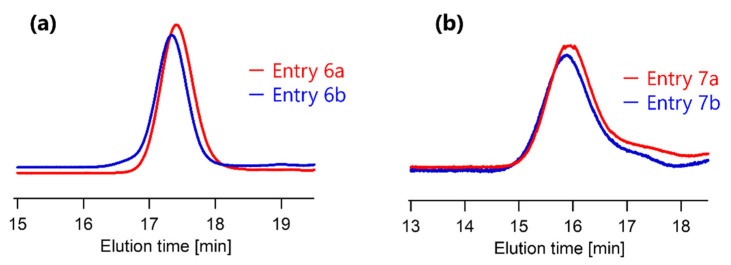
SEC chromatograms for before and after TTC removal reaction from (**a**) PSA4.9k (Entry 6a and 6b in Table 3) and (**b**) PSA17k (Entry 7a and 7b in Table 3).

Figure 3 shows SEC chromatograms before and after the TTC removal reaction from PSA4.9k and PSA17k, and Table 3 shows the number average molecular weight (*M_n_*), weight average molecular weight (*M_w_*), and polydispersity index (PDI; *M_w_*/*M_n_*) for each sample. These results show that the molecular weight and PDI were almost the same before and after the TTC removal reaction. Side reactions have often been reported, where 2 polymer radicals are coupled during removal of the RAFT-polymerized end group by radical reaction using radical initiators before the generated radical and polymer radical were coupled [18]. Our results shows that the coupling of two polymer radicals did not occur during the TTC removal reaction at 70 °C for 16 h, and the polymer main chain was not damaged from excessive amounts of isobutyronitrile radicals generated by AIBN.

**Table 3:** SEC value for PSAs.

Entry	TTC Removal Rate [%] ^a^	*M_n_* (GPC) ^b^	*M_w_* (GPC) ^b^	*M_w_*/*M_n_*^b^	Remarks
**6a**	0	4900	5200	1.05	Same as PSA4.9k
**6b**	88	5400	5600	1.04	Entry 2
**7a**	0	16,700	18,500	1.11	PSA17k
**7b**	100	17,900	19,700	1.11	Entry 5

^a^ Calculated by ^1^H-NMR. ^b^ PSt-calibrated SEC values.

#### 3.2.2. Elemental Analysis

Table 4 shows the results of elemental analysis. The theoretical mass percentage of Entries 6a and 7a were calculated from the SEC results. Entries 6b and 6c were calculated for the TTC removal rate using NMR, and Entry 7b was calculated from the estimation of the TTC removal rate as 100%. The experimental mass percentage of S for Entry 6a is slightly less than theoretical. The TTC end groups may have been removed during polymer synthesis or purification. Entry 6b is that for TTC removed from Entry 6a, the experimental mass percentage of S is significant, even if the N value is the same as the theoretical. This also confirmed that the TTC removed by the radical reaction remained with the polymer, because the *b*’ peak disappeared, whereas the *h* peaks remained in the ^1^H-NMR spectra (Figure 1). Therefore, a purification method to completely wash the removed TTC from the polymer is an issue for future work. In Entry 7b, S was not detected (or was only trace amounts below the detection limit), which indicates that TTC was completely removed. However, the mass of N was half the theoretical value, and although TTC was successfully removed, it is possible that the isobutyronitrile group derived from AIBN could not be introduced.

#### 3.2.3. MALDI-TOF MS Analysis

**Figure 4:**
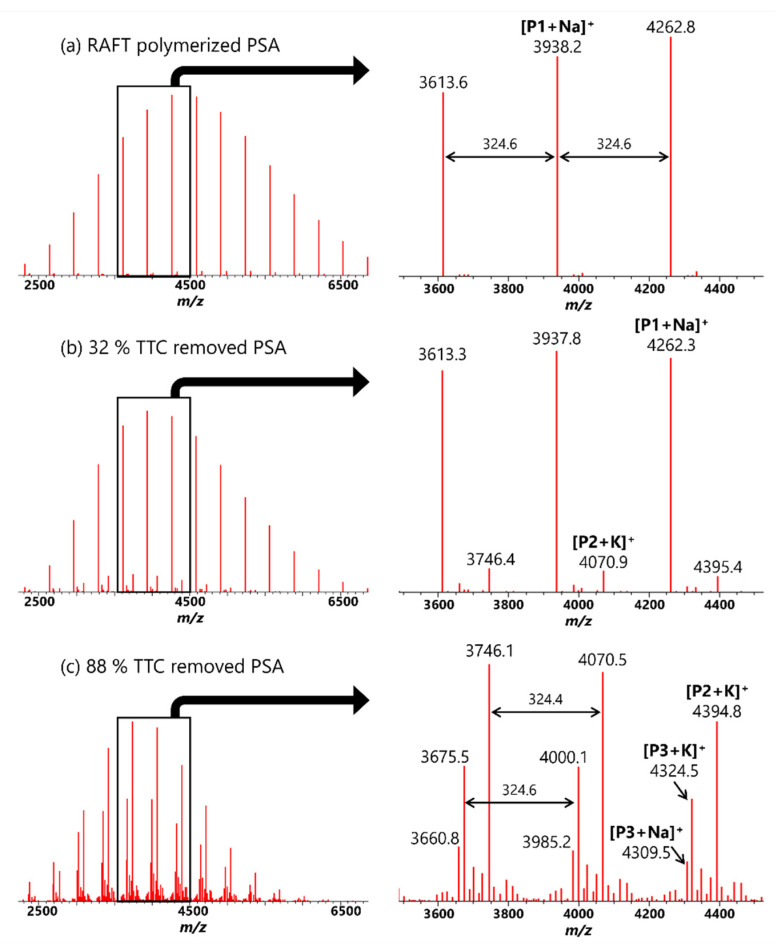
MALDI-TOF MS spectra for (**a**) RAFT polymerized PSA (PSA4.9k in Table 1), (**b**) 32% TTC removed PSA (entry 1 in Table 2, entry 6c in Table 4), and (**c**) 88% TTC removed PSA (entry 2 in Table 2, entry 6b in Table 4). The TTC removal rate was calculated from the ^1^H-NMR results.

Analysis using MALDI-TOF MS was performed to identify the end structure of PSA though the removal of TTC with an excess amount of AIBN. Figure 4 shows the MALDI-TOS MS spectra and Table 5 summarizes the various PSA chemical structures, which are speculated from the mass peaks detected by MALDI-TOF MS. The MALDI-TOS MS spec trum of the PSA structure that was synthesized by RAFT polymerization, shown in Figure 4a, revealed only single mass peaks (*m*/*z* = 3938.2, n = 11 with Na^+^), which was in agreement with the mass of PSA with TTS as the one end group and isobutyronitrile as the other (P1 in Table 5). Figure 4b shows the MALDI-TOF MS spectrum for 32% TTC removed PSA, the mass peak of which indicated isobutyronitrile at both ends of PSA (P2 in Table 5, *m/z* = 4070.9, n = 12 with K^+^), in addition to that of the [P1+Na]^+^ structure. The largely increased mass peak for isobutyronitrile at both ends [P2+K]^+^ was shown for the 88% TTC removed PSA (Figure 4c), which was prepared by optimization of the conditions for removal by AIBN, although the mass peak for TTC [P1+Na]^+^ as one end was not detected. In addition, two newly generated mass peaks (*m*/*z* = 4309.5, 4324.5) were detected, which was in good agreement with the PSA structures (shown as P3 in Table 5) with cationated ions of Na and K, respectively. There have been former reports regarding the structural change by laser effects or oxidation during MALDI measurements [24,25]; however, no peaks related with these changes were detected in the present study. From these results, it is clearly demonstrated that the isobutyronitrile group from AIBN was possibly introduced through the end group modification of PSA to remove the TTC end group due to the CTA at 70 °C for 16 h with an excess amount of AIBN.

#### 3.2.4. TGA

**Figure 5:**
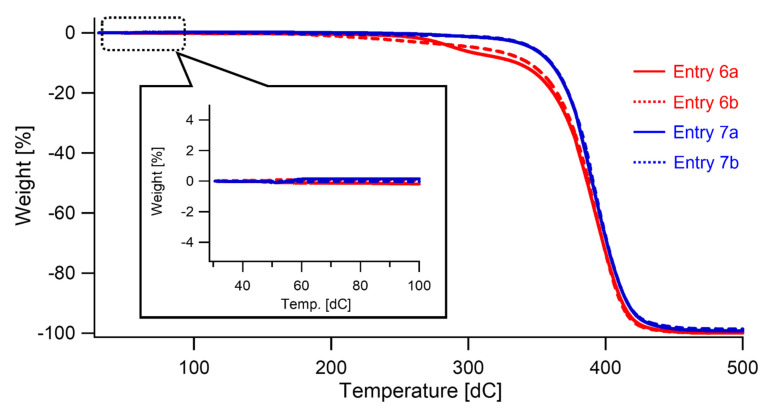
TGA curves corresponding to entries 6a, 6b, 7a, and 7b in Table 3 and Table 4 to determine the decomposition temperature of PSAs.

Figure 5 shows TGA results for entries 6a, 6b, 7a, and 7c in Table 3 and Table 4. All samples were not decomposed in the range of room temperature to 200 °C. The weight loss of entries 6a and 6b with smaller molecular weights began at around 250 °C, and then all samples were rapidly pyrolyzed at 350 °C.

#### 3.2.5. DSC Analysis

**Figure 6:**
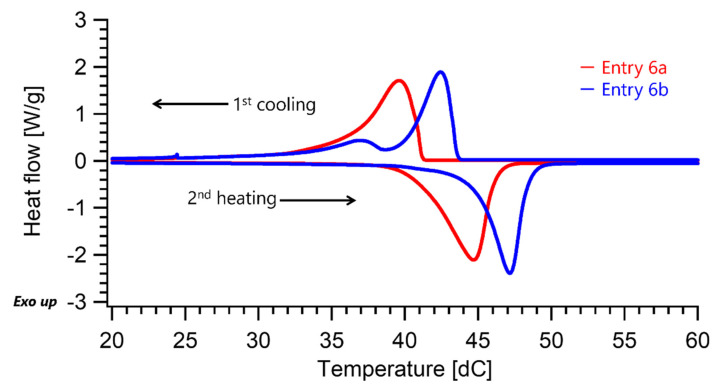
DSC curves before (red line, Entry 6a) and after TTC removal from PSA4.9k (blue line, Entry 6b). The heating/cooling rate was 5 °C/min.

Figure 6 shows two DSC curves: PSA with TTC (Entry 6a in Table 4) and PSA with the isobutyronitrile group (Entry 6b in Table 4) after modification with the respective end groups. An increase of molecular weight causes the melting point to be higher [26,27], and the crystallization temperature is dependent on the cooling speed [28]. Although these two samples have almost the same molecular weight, with the only differences being the end group of either TTC or isobutyronitrile, both the melting point and the crystallization point at the peaks were unexpectedly shifted to higher temperature by ca. 3 °C. This result suggests that the end group, long alkyl (TTC) lowers both the melting and crystallization temperatures.

Here, let us discuss the melting point temperature of the end group itself, low molecular weight CDTP, because CDTP has a long C12 alkyl chain that exhibits crystalline characteristics (melting point around 10 °C). On the other hand, the monomer of PSA, i.e., SA, also has a long C18 alkyl chain that exhibits crystalline characteristics (melting temperature around 30 °C). This melting point temperature difference of the two low molecular weight compounds is interpreted as the longer alkyl (C18, SA, monomer of PSA) has a higher melting point than that of the shorter alkyl (C12, CDTP, end group); therefore, the C12 alkyl melts and crystallizes at lower temperature. The other possible interpretation is that the end group of CDTP exists in an amorphous region instead of a crystalline region. Whichever the case, it is considered that at least the end of CDTP lowers the melting point temperature of PSA and makes the crystallite size smaller.

**Figure 7:**
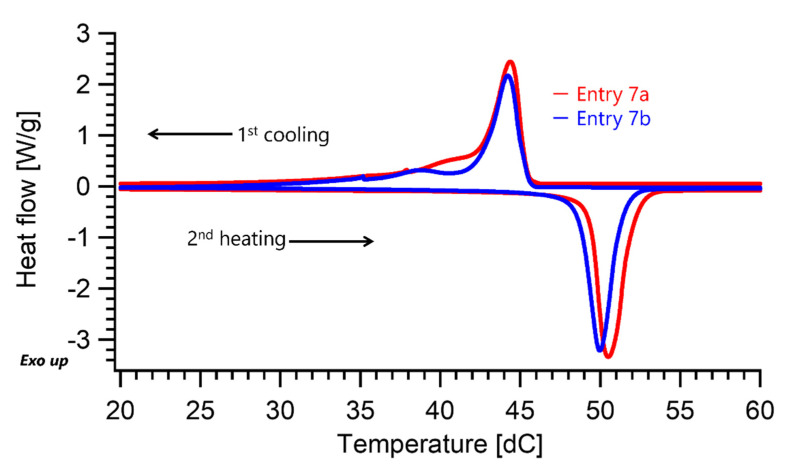
DSC curves before (red line, Entry 7a) and after TC removal from PSA17k (blue line, Entry 7b). The heating/cooling rate was 5 °C/min.

Figure 7 shows DSC curves of PSA, which has higher molecular weight, before (Entry 7a) and after (Entry 7b) the modification. A comparison of Figure 7 with Figure 6 shows that the modification makes no difference to the melting and crystallization characteristics for the larger molecular weight PSA. The volume percentage of the CDTP end group differs largely between PSA4.9k and PSA17k. In the case of PSA17k, the volume percentage of the CDTP end group is very small (ca. 2%), while that of PSA4.9k is large (ca. 7%). The effect of end group modification thus becomes negligible for PSA17k.

Here, let us consider the crystallite size through careful observation of the DSC curves in Figure 6 and Figure 7. The common point between PSA4.9k (Figure 6) and PSA17k (Figure 7) is that there are two crystallization peaks, small and large, after the removal of the end long alkyl chain, as shown in Figure 6 (6b) and Figure 7 (7b). Figure 7 (7a) shows that the lower peak is not clear and should be considered as a shoulder. DSC characteristics for a larger size crystallite indicate crystallization at higher temperature, which suggests that the higher and larger peak comes from the formation of larger size crystallites (42.4 °C in Figure 6 (6b) and 44.2 °C in Figure 7 (7b)). On the other hand, the lower and smaller peak (or shoulder) is related to the formation of smaller size crystallites (36.9 °C in Figure 6 (6b) and 38.6 °C in Figure 7 (7b)). PSA prior to modification exhibited only a single broad peak without a smaller peak or shoulder, which indicates that the end group causes a wider size distribution of crystallites, so that the peak distribution widens down to the lower temperature region. There should be no conflict regarding the interpretation before and after the modification in terms of the crystallite size and DSC curves.

Let us discuss the degree of TTC removal in detail. The removal of TTC causes the crystallite size to become larger. The second lower peak is relatively large in Entry 6b than in Entry 7b. Assuming residual TTC of ca. 12% and the volume percentage of TTC because of lower molecular weight into consideration, it can be considered that the remaining TTC causes smaller crystallite size with a larger distribution, which results in widening of the peak down to lower temperature. The DSC curve of Entry 7a shows a shoulder at lower temperature, which suggests the amount of TTC in Entry 7a is smaller than that in Entry 6a, so that the content of middle size crystallites would be less because there is less probability to disturb the crystallite growth compared with that of Entry 6a.

#### 3.2.6. XRD

**Figure 8:**
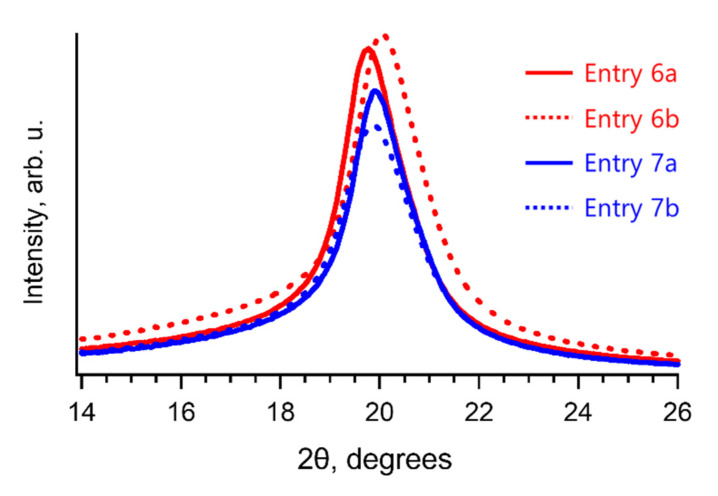
X-ray diffraction (XRD) patterns. Entry 6a (red solid line) is for PSA4.9k before TTC removal, and Entry 6b (red dashed line) is that after TTC removal. Entry 7a (blue solid line) is for PSA17k before TTC removal and Entry 7b (blue dashed line) is that after TTC removal.

Figure 8 shows the XRD patterns for PSA4.9k and PSA17k, before and after TTC removal. All the diffraction patterns were almost identical with the peak at 20° 2θ, which indicates the crystalline structure is the same because the crystalline structure of PSA is not affected by the end group. The degree of crystallinity is calculated to be ca. 50% for Entries 6a, 6b, 7a, and 7b. The crystallinity was almost the same, even if the molecular weight of PSA was different.

## 4. Conclusions

PSA was prepared by RAFT polymerization using CDTP as the CTA to examine the effect of the end group on the thermal and structural properties. The long alkyl C12 end group of PSA polymerized by the RAFT process was removed by a radical reaction using AIBN. The reaction condition was optimized, especially with respect to the temperature, which resulted in the successful removal of TTC, which was characterized by ^1^H-NMR, elemental analysis, and SEC. The polymer structures before and after removal of TTC were determined by MALDI-TOF MS spectra. We demonstrated removal TTC and modifying isobutyronitrile end group were succeeded by using AIBN. The thermal and structural properties were characterized using TGA, DSC, and XRD, which provided evidence that the long alkyl end groups of TTC lower the melting point and crystallization peak temperatures by 3 °C, which in turn affects the formation of different crystallite sizes with identical crystalline structure and degree of crystallinity.

## Figures and Tables

**Table 1:** RAFT polymerization of SA with CDTP as CTA.

Polymer	[SA]_0_/[CDTP]_0_/ [AIBN]_0_ ^a^	Conv. [%] ^b^	*M_n_*(theo.) ^c^	*M_n_*(SEC) ^d^	*M_w_*(SEC) ^d^	*M_w_*/*M_n_*^d^
PSA4.9k	45/3/1	85	4500	4900	5200	1.05
PSA17k	300/3/1	78	26,000	16,700	18,500	1.11

^a^ Solution polymerization with toluene (2M). ^b^ Calculated by ^1^H-NMR. ^c^ Theoretical Mn calculated with [SA]_0_, [CDTP]_0_, and monomer conversion. ^d^ PSt-calibrated SEC values.

**Table 2:** TTC removing reaction from RAFT-polymerized PSA.

Entry	Precursor	Removal Condition Using Precursor				
		[AIBN]_0_/[PSA]_0_	Temp. [°C]	Time [h]	AIBN Decomposition Rate [%] ^a^	TTC Removal ^b^
**1**	PSA4.9k	30/1	65 °C	24 h	81.1	32%
**2**		30/1	70 °C	16 h	88.9	88%
**3**		30/1	80 °C	2.5 h	72.2	none
**4**		30/1	90 °C	3 h	99.5	none
**5**	PSA17k	30/1	70 °C	16 h	88.9	N/A

^a^ Calculated data. ^b^ Calculated by ^1^H-NMR in Figure 1.

**Table 4:** Results of elemental analysis for PSAs.

Entry	TTC Removal Rate [%] ^a^	C [%] (theo. ^b^)	H [%] (theo. ^b^)	N [%] (theo. ^b^)	S [%] (theo. ^b^)	Remarks
**6a**	0	76.2 (76.4)	12.4 (12.2)	0.3 (0.3)	1.7 (1.9)	PSA4.9k in Table 1
**6b**	88	77.0 (76.8)	12.2 (12.2)	0.4 (0.4)	1.2 (1.3)	Entry 2 in Table 2
**6c**	32	76.7 (77.4)	12.2 (12.3)	0.6 (0.6)	0.4 (0.2)	Entry 1 in Table 2
**7a**	0	77.6 (77.3)	12.4 (12.4)	0.1 (0.2)	0.5 (0.5)	PSA17k in Table 1
**7b**	100	77.4 (77.7)	12.4 (12.4)	0.1 (0.2)	0.0 (0.0)	Entry 5 in Table 2

^a^ Calculated by ^1^H-NMR. ^b^ Calculated by SEC value and TTC removal rate by ^1^H-NMR.

**Table 5:** Polymer structures corresponding to the peaks in Figure 4.

Polymer	Structure	n	Cationization	*m*/*z*	
				Theoretical	Experimental
**P1**	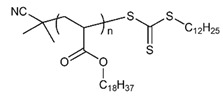	11	Na	3938.6	3938.2
		12	Na	4263.1	4262.3
**P2**	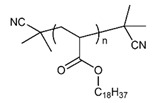	12	K	4069.8	4070.9
		13	K	4394.3	4394.8
**P3**	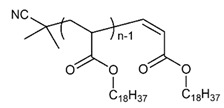	13	Na	4309.1	4309.5
		13	K	4325.2	4324.5

## Data Availability

The raw data presented in this study are available on request from the corresponding author.

## References

[1] Xia Y., Nicholas A.D., Burke N.A.D., Stolver H.D.H. (2006). End Group Effect on the Thermal Response of Narrow-Disperse Poly(N-isopropylacrylamide) Prepared by Atom Transfer Radical Polymerization. Macromolecules.

[2] Kujawa P., Segui F., Shaban S., Diab C., Okada Y., Tanaka F., Winnik F.M. (2006). Impact of End-Group Association and Main-Chain Hydration on the Thermosensitive Properties of Hydrophobically Modified Telechelic Poly(N-isopropylacrylamides) in Water. Macromolecules.

[3] Roth P.J., Jochum F.D., Forst F.R., Zentel R., Theato P. (2010). Influence of End Groups on the Stimulus-Responsive Behavior of Poly[oligo(ethylene glycol) methacrylate] in Water. Macromolecules.

[4] Jochum F.D., Borg L., Roth P.J., Theato P. (2009). Thermo- and Light-Responsive Polymers Containing Photoswitchable Azobenzene End Groups. Macromolecules.

[5] Chiefari J., Chong Y.K., Ercole F., Krstina J., Jeffery J., Le T.P.T., Mayadunne R.T.A., Meijs G.F., Moad C.L., Moad G. (1998). Living Free-Radical Polymerization by Reversible Addition-Fragmentation Chain Transfer: The RAFT Process. Macromolecules.

[6] Moad G., Rizzardo E., Thang S.H. (2005). Living Radical Polymerization by the RAFT Process. Aust. J. Chem..

[7] Moad G., Rizzardo E., Thang S.H. (2009). Living Radical Polymerization by the RAFT Process—A Second Update. Aust. J. Chem..

[8] Moad G., Rizzardo E., Thang S.H. (2012). Living Radical Polymerization by the RAFT Process—A Third Update. Aust. J. Chem..

[9] Keddie D.J., Moad G., Rizzardo E., Thang S.H. (2012). RAFT Agent Design and Synthesis. Macromolecules.

[10] Moad G., Chong Y.K., Postma A., Rizzardo E., Thang S.H. (2005). Advances in RAFT polymerization: The synthesis of polymers with defined end-groups. Polymer.

[11] Harrisson S. (2009). Radical-Catalyzed Oxidation of Thiols by Trithiocarnate and Dithioester RAFT Agents: Implications for the Preparation of Polymers with Terminal Thiol Functionality. Macromolecules.

[12] Yu B., Chan J.W., Hoyle C.E., Lowe A.B. (2009). Sequential Thiol-Ene/Thiol-Ene and Thiol-Ene/Thiol-Yne Reactions as a Route to Well-Defined Mono and Bis End-Functionalized Poly(N-isoprolacrylamide). J. Polym. Sci. A Polym. Chem..

[13] Postma A., Davis T.P., Evans R.A., Li G., Moad G., O’Shea M.S. (2006). Synthesis of Well-Defined Polystyrene with Primary Amine End Groups through the Use of Phthalimid-Functional RAFT Agents. Macromolecules.

[14] Dietrich M., Glassner M., Gruendling T., Schmid C., Falkenhagen J., Barner-Kowollik C. (2010). Facile conversion of RAFT polymers into hydroxyl functional polymers: A detailed investigation of variable monomer and RAFT agent combinations. Polym. Chem..

[15] Gruendling T., Pickford R., Guilhaus M., Barner-Kowollik C. (2008). Degradation of RAFT Polymers in a Cyclic Ether Studied via High Resolution ESI-MS: Implications for Synthesis, Storage, and End-Group Modification. J. Polym. Sci. A Polym. Chem..

[16] Chong Y.K., Moad G., Rizzardo E., Thang S.H. (2007). Thiocarbonylthio End Group Removal from RAFT-Synthesized Polymers by Radical-Induced Reduction. Macromolecules.

[17] Perrier S., Takolpuckdee P., Mars C.A. (2005). Reversible Addition-Fragmentation Chain Transfer Polymerization: End Group Modification for Functionalized Polymers and Chain Transfer Agent Recovery. Macromolecules.

[18] Chen M., Moad G., Rizzardo E. (2009). Thiocarbonylthio End Group Removal from RAFT-Synthesized Polymers by a Radical-Induced Process. J. Polym. Sci. A Polym. Chem..

[19] Willcock H., O’Reilly R.K. (2010). End group removal and modification of RAFT polymers. Polym. Chem..

[20] Moad G., Rizzardo E., Thang S.H. (2011). End-functional polymers, thiocarbonylthio group removal/transformation and reversible addition-fragmentation-chain transfer (RAFT) polymerization. Polym. Int..

[21] Miho Y., Hirai S., Nakano R., Sekiguchi H., Yao S. (2018). Modification of polyethylene using side-chain crystalline block copolymer and evaluation of hydrophilicity. Polym. J..

[22] Wang S., Kesava S.V., Gomez E.D., Robertson M.L. (2013). Sustainable Thermoplastic Elestmers Derived from Fatty Acids. Macromolecules.

[23] Yokota T., Inoue Y., Terakawa Y., Reeder J., Kaltenbrunner M., Ware T., Yang K., Mabuchi K., Murakawa T., Sekino M. (2015). Ultraflexible, large-area, physiological temperature sensors for multipoint measurements. Proc. Natl. Acad. Sci. USA.

[24] Favier A., Ladaviere C., Charreyre M.T., Pichot C. (2004). MALDI-TOF MS Investigation of the RAFT Polymerization of a Water-Soluble Acrylamide Derivative. Macromolecules.

[25] Xu J., He J., Fan D., Wang X., Yang Y. (2006). Aminolysis of Polymers with Thiocarbonylthio Termini Prepared by RAFT Polymerization: The Difference between Polystyrene and Polymethacrylates. Macromolecules.

[26] Jeon J., Lee H.B.-R., Bao Z. (2013). Flexible Wireless Temperature Sensors Based on Ni Microparticle-Filled Binary Polymer Composites. Adv. Mater..

[27] Kim B., Mather P.T. (2002). Amphiphilic Telechelics Incorporating Polyhedral Oligosilsesquioxane: 1. Synthesis and Characterization. Macromolecules.

[28] Schick C. (2009). Differential scanning calorimetry (DSC) of semicrystalline polymers. Anal. Bioanal. Chem..

